# Obstacles and solutions for implementing amyloid‐targeting treatments in Europe

**DOI:** 10.1002/dad2.70367

**Published:** 2026-07-01

**Authors:** Anna Hofmann, Robert Perneczky

**Affiliations:** ^1^ Department of Neurology and Knight Alzheimer Disease Research Center Washington University St. Louis Missouri USA; ^2^ German Network for Memory Clinics (DNG) Aachen Germany; ^3^ Department of Psychiatry and Psychotherapy LMU Hospital Munich Germany; ^4^ German Center for Neurodegenerative Diseases (DZNE) Munich Germany; ^5^ Munich Cluster for Systems Neurology (SyNergy) Munich Germany; ^6^ Ageing Epidemiology (AGE) Research Unit, School of Public Health Imperial College London London UK; ^7^ Division of Neuroscience University of Sheffield Sheffield UK

**Keywords:** Alzheimer's disease blood‐biomarker tests, clinical registries, digital cognitive testing, European health policy makers and regulatory agencies, feasibility and safety of amyloid‐targeting treatments

## Abstract

Recent approvals of disease‐modifying therapies by the European Medicines Agency mark a historic shift in the treatment landscape of Alzheimer's disease (AD) within the European Union that will challenge national health‐care systems and require major adaptations and modernization. This Perspective article provides an overview of the major obstacles in Europe concerning successful implementation of amyloid‐targeting treatments and offers potential solutions to overcome them. Major hurdles include a lack of recognition regarding the critical importance of an early, biomarker‐based AD diagnosis; low acceptance of blood tests and digital cognitive screening tools; insufficient investment in magnetic resonance imaging capacities; and a fragmented infrastructure for clinical registries. We call on European clinicians, research institutions, and policy makers for a bold and coordinated action to urgently modernize diagnostic pathways and monitoring infrastructure to deliver novel AD treatments in a timely, safe, and equitable manner to all patients who may benefit.

## Highlights

- European Medicines Agency approval of amyloid‐targeting treatments represents a historic shift in the European Union.
- Successful clinical implementation requires major health‐care system adjustments.
- Europe must invest in accessible, scalable diagnostics and harmonized registries.

## 1 EUROPE AT A CROSSROADS: EMBRACING THE ERA OF NEW ALZHEIMER'S DISEASE TREATMENTS

The recent marketing authorization by the US Food and Drug Administration (FDA) of lecanemab in 2023 and donanemab in 2024 marks a historic inflection point in the field of Alzheimer's disease (AD) therapies. These amyloid‐targeting treatments (ATTs) represent a dramatic shift to biological targeting of an underlying disease mechanism. Compared to the United States (US), Europe's regulatory and implementation trajectory has been more fragmented. The European Medicines Agency (EMA) initially rejected the approval of lecanemab, prompting widespread criticism from experts.^1^ A reversal came in April 2025, when the EMA recommended marketing authorization for lecanemab in the European Union (EU). Likewise, after initial rejection, the European Commission finally granted marketing authorization for donanemab in July 2025. Thus, just as in the United States, these drugs are now approved for the treatment of early symptomatic AD, that is, the clinical disease stages of mild cognitive impairment (MCI) or mild dementia.

Even after regulatory approval, the European landscape remains heterogeneous with regards to treatment availability due to a complex benefit assessment and price negotiation process through separate national institutions. For instance, Germany plays a pioneering role as, here, EMA‐approved drugs are preliminarily covered at list price for 6 months and therefore accessible almost immediately. During this time period, the *Gemeinsame Bundesausschuss* (GBA), based on a report by the *Institut für Qualität und Wirtschaftlichkeit im Gesundheitswesen*, decides about the expectable benefit of this new therapy in addition to the current standard medication for the German patient population specifically. For lecanemab, this judgment was rendered in December 2025, concluding that there is no additional benefit. On this basis, the GBA has now initiated negotiations with health insurance companies to determine the final market price under the *Arzneimittelmarktneuordnungsgesetz*. If this process results in a decision against regular reimbursement or a significantly lower market price than intended, the manufacturer may withdraw lecanemab from the German market. An according decision for donanemab is still pending. Likewise, in France, the *Haute Autorité de Santé* has refused to grant early access reimbursement, too, as the drug's benefit did not seem clinically significant enough facing the financial costs and potential therapy‐related risks. Similarly, in the UK, lecanemab was approved in 2024 by the Medicines and Healthcare products Regulatory Agency, but the National Institute for Health and Care Excellence has not recommended it for routine use within the National Health System (NHS) due to concerns about its cost effectiveness. Thus, both lecanemab and donanemab are now technically available in selected EU countries, including Germany and Austria; however, practical access is still limited and might be threatened fundamentally in the nearer future. Further, in Spain, rollout is not expected before autumn 2026. In Switzerland, decisions by Swissmedic on both anti‐amyloid drugs are still pending. For most Eastern European countries, ATT availability is entirely questionable within the upcoming years.

This uneven landscape reflects deeper systemic challenges. While the scientific community has entered a new era, Europe's health‐care systems remain constrained by infrastructural fragmentation and policy inertia. If unaddressed, these barriers risk delaying equitable access to transformative therapies and, thus, increasing existing health‐care disparities across Europe. A comparable situation of no (full) reimbursement due to lack of assessed added value despite existing EMA approval can also be observed for certain novel cancer therapies, leading to health‐care disadvantages for different Eastern European countries.^2^

This Perspective article calls for a bolder, harmonized European strategy, one that reimagines diagnostic pathways and integrates scalable technologies to ensure timely access to disease‐modifying treatments for all patients that could benefit.

## 2 FEASIBILITY OF ANTI‐AMYLOID TREATMENT: A GLOBAL IMPLEMENTATION CHALLENGE

Initial real‐world experience with lecanemab in the US demonstrates that ATT is clinically feasible and can be administered safely, but primarily by highly specialized, resource‐rich academic centers. Also, initial patients tend to come from privileged backgrounds.^3^ This early success highlights another critical disparity: the infrastructure required for safe and effective ATT delivery is far from universally available.

ATT eligibility hinges on a biologically confirmed AD diagnosis, until recently based on either cerebrospinal fluid (CSF) testing or amyloid positron emission tomography (PET). In May 2025, the FDA approved their first AD blood test (Fujirebio Lumipulse phospho‐tau217/Aβ1‐42 plasma ratio), offering a potentially scalable alternative. Also, in October 2025, the FDA approved the Roche Elecsys p‐tau181 blood test to rule out AD in the primary care setting. Within Europe, the Roche Elecsys p‐tau181 blood test recently achieved a *Conformité Européenne* (CE) mark to rule out AD pathology, too.^4^ Further, the PrecivityAD2 blood test has already received both a CE mark and UK medical device certification, enabling its commercial use in evaluating patients with MCI or dementia.^5^, ^6^ However, before ATT are initiated, there are other prerequisites that further increase the complexity of implementation including initial neurocognitive testing, baseline magnetic resonance imaging (MRI), apolipoprotein E (*APOE*) genotyping to assess amyloid‐related imaging abnormality (ARIA) risk, in‐depth patient counseling about expected risks and benefits, and a shared decision‐making process regarding treatment initiation.

Per prescribing information, lecanemab treatment requires intravenous infusions every 2 weeks over 18 months, with surveillance MRI scans for ARIA screening recommended at multiple time points (e.g., prior to the third, fifth, seventh, fourteenth, and twenty‐sixth doses).^7^ Subsequently, the label allows for either continued biweekly dosing or transition to monthly dosing after 18 months of treatment. For donanemab, infusions are required every month and monitoring MRIs should be performed prior to the second, third, fourth, and seventh infusion.^8^ According to appropriate use recommendations,^9^ it is reasonable to consider stopping therapy based on a negative follow‐up amyloid PET scan, typically obtained 12 to 18 months after treatment start. Subsequently, individuals likely remain below the threshold for amyloid positivity for several years.^10^ In settings in which PET is not available to monitor treatment response, clinicians may choose to limit the duration of donanemab treatment to ≈ 18 months, based on clinical trial data^11^ indicating that > 75% of patients achieved amyloid clearance, as measured by PET, within that timeframe.

For both substances, ongoing clinical and cognitive assessments are also essential throughout treatment, typically every 6 months. Finally, patient‐related data entry into clinical registries such as ALZ‐NET^12^ may be mandatory and require additional workload. Although only a small portion of patients with early symptomatic AD is expected to be ATT eligible,^13^ these requirements will place substantial demands on health‐care systems for financial resources, staffing, coordination between memory and infusion clinics, and neuroradiological expertise. The burden is particularly acute in settings with limited infrastructure or workforce capacity.

Hence, there is an urgent need for pragmatic strategies to decentralize and streamline ATT delivery. The recent FDA approval of subcutaneous lecanemab for maintenance dosing is a first important step into that direction. However, further relevant aspects include facilitating access to blood tests, increasing imaging capacities, leveraging digital, scalable tools for cognitive screening and monitoring, as well as expanding participation of non‐academic settings. Without such adaptations, the promise of ATT will remain out of reach for many patients.

## 3 EARLY DIAGNOSIS: A PREREQUISITE STILL UNDER DEBATE

Early diagnosis of AD has become essential with the clinical availability of ATT. In response, the US‐based Alzheimer's Association Workgroup proposed revised diagnostic criteria emphasizing biomarker‐based definitions, effectively shifting AD from a syndromic to a biological construct.^14^ In contrast, the European International Working Group maintains a clinical–biological definition, requiring the presence of cognitive symptoms alongside biological evidence.^15^ This distinction remains reasonable under current regulatory frameworks as ATT are indicated for individuals with at least very mild AD symptoms (Clinical Dementia Rating [CDR] global score of 0.5 or 1). However, this conceptual paradigm may soon be outdated. Ongoing clinical trials are exploring the efficacy of ATT in delaying onset of symptomatic AD in cognitively unimpaired individuals with evidence of AD pathology.^16^, ^17^ Should these trials demonstrate benefit, the field will need to adopt a purely biological definition of AD also in clinical practice—one that enables intervention before clinical decline becomes apparent. Against this backdrop, traditional approaches emphasizing the “right not to know” about a potential AD diagnosis particularly at the MCI stage and questioning the ability of biomarkers to confirm AD before present symptoms of full‐blown dementia are clearly hindering progress.^18^ Such positions may be appropriate for individual patients but are unethical on a broader level as they delay diagnosis and treatment in patients who may benefit most from ATT.

As the field moves toward earlier and more precise intervention, clinical guidelines must evolve accordingly—grounded in science, rather than concerns about stigma, which may worsen outcomes.

## 4 USE OF BLOOD BIOMARKERS: FROM PROMISE TO PRACTICE

As ATT requires confirmation of underlying amyloid pathology, a rapid biomarker‐based diagnosis of AD has become critically important. Traditionally, two different biomarker modalities have been used in the clinic, that is, either amyloid PET or CSF tests.

Amyloid PET offers significant advantages, both at baseline and in response to ATT, including the ability to display regional distribution of amyloid deposition in the brain and quantification through the Centiloid (CL) scale. For instance, taking into account the mean rate of amyloid clearance observed in clinical trials (≈ 60 CL/year), the baseline CL value, if available, may assist in determining an appropriate timepoint for follow‐up imaging and deciding on potential discontinuation of therapy. However, PET imaging also bears the risk of radiation and demands highly specialized equipment and personnel. In the US, amyloid PET has been reimbursed by Medicare since 2023 and is regularly available, even if almost only at major academic medical centers. In contrast, access remains quite limited across much of Europe. For example, in Germany, despite a well‐resourced health‐care system, amyloid PET scans are rarely reimbursed and performed at low volumes by international standards.

CSF tests are broadly available in Europe; however, they also have significant drawbacks limiting their scalability including invasiveness as well as burden to patients and providers. Also, not all patients are suitable candidates for a lumbar puncture, including those with former back surgeries, taking anticoagulants, or certain central nervous system disorders affecting CSF circulation such as normal pressure hydrocephalus that can falsify fluid biomarker concentrations.^19^ This diagnostic bottleneck may threaten successful ATT rollout in Europe.

Blood biomarkers offer a transformative opportunity. Compared to PET imaging and CSF analysis, they have only minimal procedural risk; also, they are less labor intensive and more accessible. Thus, their scalability and affordability make them ideally suited for broader deployment, particularly in primary care and under‐resourced settings. Moreover, their routine clinical implementation will likely reduce the time until diagnosis and treatment start, which is critical for a medication that should be initiated in incipient disease stages and has been shown to delay progression by ≈ 6 months over 18 months of treatment.^11^, ^20^

Recent advances have yielded highly accurate AD blood tests, especially those measuring phosphorylated tau (pTau)‐217, that perform equivalently in detecting AD pathology compared to CSF and amyloid PET with sensitivities and specificities of ≈ 90%.^21^, ^22^ It will be essential that this matter of fact becomes internalized by physicians diagnosing patients with AD. Notably, not every patient is equally suited for blood biomarker testing, particularly those with relevant comorbidities such as chronic kidney disease.^23^ Until their complete validation and routine use in everyday clinical practice, some AD blood tests, for example, in patients with a low pretest probability (due to young age or atypical syndrome) or intermediate results, may still require confirmation with a traditional biomarker modality. However, it should be acknowledged that also CSF and amyloid PET imaging, traditionally considered the “gold standard” in AD diagnosis, are not infallible, either. CSF and amyloid PET deliver discordant results in ≈ 10% of clinical cases.^24^ Amongst others, this is due to evident differences in the pathobiological processes they reflect, that is, the lifetime accumulation of insoluble amyloid versus the equilibrium of certain fluid analytes at a given timepoint. As blood essentially reflects CSF biomarker changes, it is expected that AD blood test and amyloid PET results are discordant to a similar extent. Thus, every biomarker modality should be selected and interpreted carefully within the individual clinical context. Although blood biomarkers cannot yet be used for treatment monitoring on the individual level due to weak correlations with amyloid PET (CL values),^9^ they are uniquely positioned to dramatically reduce the need for PET scans and lumbar punctures during initial determination of treatment eligibility.^25^, ^26^ There exists already first evidence for high diagnostic accuracy of AD blood tests even in primary and secondary care settings^27^ as well as heterogeneous memory clinic cohorts.^28^

Accordingly, several recent US and international guidelines recommend the use of such highly accurate blood biomarker tests to confirm the diagnosis of AD.^9^, ^26^, ^29^ An EU/US task force has already outlined a diagnostic roadmap for integration of AD blood biomarkers, aiming to reduce unnecessary confirmatory procedures.^30^ Further, the recently published French appropriate use recommendations for lecanemab already consider the usage of AD blood tests as appropriate for determination of ATT eligibility, provided that a highly precise assay including pTau‐217 and a two cut‐off approach are being used.^31^ Despite already existing regulatory clearances outlined above, a lack of reimbursement is still limiting the uptake of AD blood tests even within the US. In Europe, real‐world implementation of these novel tools is almost negligible. This disconnect may be driven by persistent skepticism regarding the clinical utility of blood biomarkers.

However, multiple international, mostly European‐based, initiatives are actively driving forward the validation and implementation of blood biomarker‐based approaches in real‐world settings (Table 1). The Davos Alzheimer's Collaborative is building diverse global cohorts to support early diagnosis using blood and digital biomarkers. EU‐funded projects such as PREDICTOM, PROMINENT, ACCESS‐AD, AD‐RIDDLE,^32^ and others are developing web‐based platforms that integrate cognitive screening with biomarker data to enable personalized risk assessment and early intervention. The recently launched ADAPT trial will investigate whether application of an AD blood biomarker test leads to a faster and more reliable diagnosis in > 1000 patients from across the UK compared to standard of care, which is a purely clinically based diagnosis in > 90% of patients.

**TABLE 1:** Overview of different European initiatives on AD blood biomarker implementation in real‐world settings.

Initiative	Scientific and clinical goals
**Davos Alzheimer's Collaborative**	Align health‐care systems worldwide, especially including those in low‐ and middle‐income countries Establishment of several, ethno‐racially diverse cohorts for the collection of new data including blood biomarkers and digital cognitive assessments Enable accurate and early diagnosis of AD globally
**PROMINENT (Precision Medicine in Neurodegeneration)**	Pan‐European initiative leveraging biomarker and digital tools Improve and individualize diagnosis and treatment of neurodegenerative diseases
**PREDICTOM**	Online biomarker and cognitive screening approach for early and accurate diagnosis of AD Develop a screening platform capable of identifying people at risk of dementia, before the first symptoms appear
**AD‐RIDDLE**	Digital community portal comprising blood biomarker test results and self‐guided cognitive assessment tools Paving the way to personalized lifestyle interventions and referral to appropriate health‐care resources
**ACCESS‐AD**	Translation of diagnostic and monitoring innovations (blood and imaging biomarkers, digital and AI tools) into real‐world practice in a coordinated and equitable way with the overall goal to improve AD patient outcomes across Europe
**ADAPT**	Clinical trial across the UK in > 1000 participants from ethno‐racially diverse backgrounds Investigating whether blood testing in clinical practice leads to a faster and more reliable diagnosis and better care for AD patients

Abbreviation: AD, Alzheimer's disease.

To realize the full potential of ATT, Europe must accelerate the clinical integration of blood biomarkers. This will require not only a regulatory and reimbursement reform, but also a cultural shift among clinicians and policy makers toward embracing scalable, evidence‐based innovation.

## 5 DIGITAL BIOMARKERS AND COGNITIVE SCREENING: UNLOCKING SCALABLE EARLY DETECTION

ATT are approved for AD patients with either MCI or mild dementia. Thus, precise neuropsychological assessment will be key for the timely identification of eligible individuals. However, access to neuropsychological services is limited across Europe due to skilled workforce shortages. As a much less labor‐intensive alternative, digital cognitive assessments are rapidly emerging as a critical complement to biological markers in the early detection of AD. These tools either adapt existing paper and pencil–based cognitive tests to digital versions or develop novel digital assessments, which are delivered via web platforms, tablets, or mobile apps that allow remote self‐administration (Table 2). Thus, they offer low‐cost and user‐friendly solutions that can be deployed in community settings, primary care, and even directly in patients’ homes. Their potential lies not only in accessibility but also in scalability and velocity: digital assessments can be administered repeatedly, enabling dynamic and longitudinal tracking of cognitive changes over time. Even beyond cognitive screening itself, passive digital biomarkers derived from the corresponding task completion, such as speech analysis, eye tracking, smartphone and digital pen usage patterns, can be integrated into artificial intelligence (AI)‐driven platforms for risk stratification and prognostic modeling. However, these tools still vary in their degree of commercialization and validation against other AD biomarkers. Further, their integration into diagnostic workflows requires not only technical validation and clinician training, but also regulatory recognition and reimbursement pathways.^33^, ^34^ For example, in Germany, neurocognitive testing is currently only reimbursed if provided by a specialist physician or certified neuropsychologist.^35^ Hence, despite their promise, digital cognitive tools remain mostly underused in clinical practice, although according to a recent survey, some of them already show a high level of acceptance by patients and physicians likewise.^36^

**TABLE 2:** Overview of selected digital and remote cognitive testing tools. Columns provide additional information on context of use and evaluation, mode of application, and specificity for AD versus dementia generally.

Testing tool	Context of use	Digital versus remote	Specificity to AD
**Cogstate**	Particularly suited for early detection	Digital	High, used in several ongoing research studies such as the Dominantly Inherited Alzheimer's Network (DIAN)‐Trials Unit
**eMOCA**	Digitized Montreal Cognitive Assessment (MoCA) version, identical to paper and pencil version, assessment by clinician	Digital (tablet)	General detection of cognitive impairment, not AD‐specific
**eCDR** ^51^	Validated against standard CDR assessment, not widely accepted in use even in research‐context	Digital	General detection of cognitive impairment, not AD specific
**Ambulatory Research in Cognition (ARC)** ^52^	Included in several observational study cohorts (DIAN, DELCODE, and BioFINDER‐2)	Remote (app)	General detection of cognitive impairment, not AD specific
**BraincheX** ^53^	Language‐independent; currently being deployed across German community settings as well as primary and secondary care practices	Remote	General detection of cognitive impairment, not AD specific
**NeotivCare**	Neuroanatomically informed; research use	Remote (app)	Early differentiation between age‐related changes and cognitive decline associated with neurodegeneration, but not AD specific
XpressO^54^	Self‐administered MoCA version, validated against eMoCA	Remote	Like eMoCA
**BioCog**	Validated in primary care and in combination with blood biomarker tests	Remote	High accuracy in detecting clinical AD, especially when applied using a two‐cut‐off approach
**BrainTest**	Digitized version of the Self‐Administered Gerocognitive Exam (SAGE)	Remote (tablet)	General detection of cognitive impairment, not AD specific

Abbreviation: AD, Alzheimer's disease.

**FIGURE 1:**
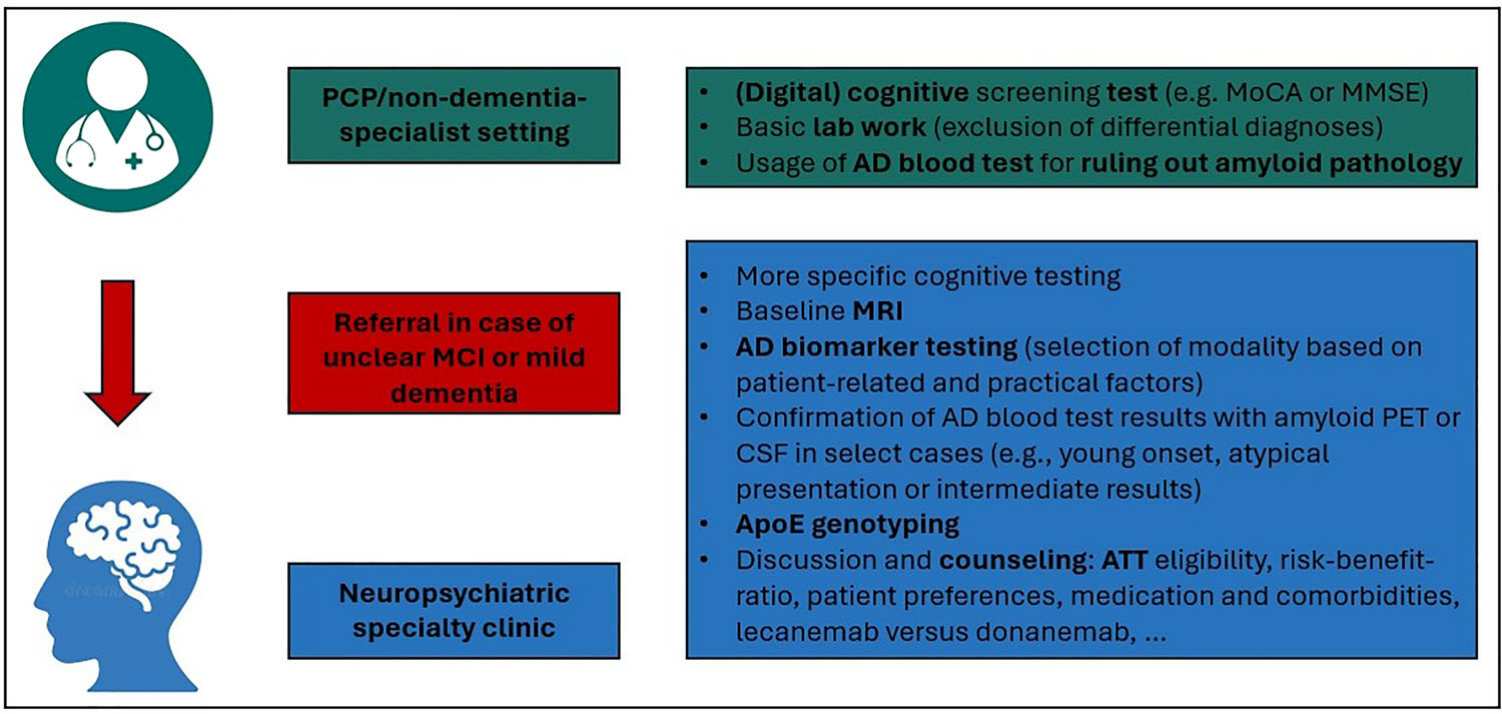
Patient flow along the AD diagnostic process and determination of ATT eligibility. AD, Alzheimer's disease; *APOE*, apolipoprotein E; ATT, amyloid‐targeting treatment; CSF, cerebrospinal fluid; MCI, mild cognitive impairment; MMSE, Mini‐Mental State Examination; MoCA, Montreal Cognitive Assessment; MRI, magnetic resonance imaging; PET, positron emission tomography; PCP, primary care physician.

Several European initiatives, such as the already mentioned Davos Alzheimer's Collaborative, PROMINENT, and AD‐RIDDLE, are further actively validating digital cognitive tools not only in different research cohorts but also real‐world settings. The harmonization of digital assessment protocols across countries and care settings will be essential to ensure consistency and comparability. In context of Europe's fragmented diagnostic infrastructure, digital biomarkers offer a unique opportunity to democratize access to early AD detection, serving as the front line of a tiered diagnostic ecosystem. For instance, combination of the digital BioCog assessment with a blood biomarker test has proven to allow the diagnosis of AD with high accuracy even in primary care.^37^ Thus, especially when paired with blood biomarkers, digital tools may form a powerful diagnostic duo, capable of identifying patients at risk for AD in its earliest stages and triaging those for further evaluation, thereby significantly reducing the need for expensive and invasive procedures as well as alleviating burden on memory clinics, finally reducing diagnostic delays, and accelerating access to disease‐modifying therapies.^38^ These goals might be further supported by integrative approaches such as the British Kneu Health app,^39^ whereby patients seeking dementia specialist appointments are being pre‐classified not only based on their digital cognitive test results, but also further demographic and clinical details. Such scalable tools significantly reduce the burden associated with reviewing extensive and unstructured medical referral paperwork, and automatically prioritize patients who may be eligible for ATT. Figure 1 illustrates patients’ flow along the AD diagnostic process combining digital cognitive screening with subsequent (blood) biomarker testing aiming at determination of ATT eligibility.

## 6 ECONOMIC ASPECTS: TOWARD COST‐EFFICIENT AND FAST DIAGNOSTIC PATHWAYS

The EU spends 10% of its gross domestic product on health care, with countries like Germany, Switzerland, Austria, France, and Sweden being particularly high spending, and Germany consistently setting the record for highest health‐care expenditures among EU countries with almost €500 billion annually.^40^ Dementia care is a relevant economic burden in Europe, with significant variation by region: mean individual annual costs vary from almost €8,000 in Eastern Europe and the Baltics to > €70,000 in the British Isles.^41^

Within the United States, both lecanemab and donanemab have yearly manufacturer‐set list prices of ≈ $30,000. However, when including necessary ancillary costs such as PET scans, MRIs for monitoring, and infusion services, the total yearly costs can rise to almost $100,000 per patient. Although ATT undoubtedly benefit some AD patients,^11^, ^20^ high treatment costs also lead to the valid and complex question about cost effectiveness of these medications. As outlined above, this question has already been denied by paying institutions in some even rather wealthy European countries while the debate is still ongoing in several other states.

In this context, the convergence of blood biomarkers and digital cognitive assessments offers not only a clinical promise but also a compelling economic opportunity. Traditional tools, relying on in‐person paper and pencil–based neuropsychological assessments as well as CSF analysis or amyloid PET imaging, are resource intensive, invasive, and often inaccessible outside of academic centers. These limitations are not only logistical but economic: In particular, the widespread reliance on amyloid PET and lumbar punctures would impose unsustainable costs on national health‐care systems, particularly in countries with aging populations and constrained budgets.

According to such a recent model calculation for Germany over the next 20 years, projected wait times for the diagnostic process to determine ATT eligibility could reach ≈ 50 months under current conditions, which include very limited amyloid PET capacity. However, if an AD blood test is used as an additional triage step in primary care, this wait time could be reduced to < 25 months. Despite this improvement, a waiting time still exceeding 2 years until treatment initiation would likely result in avoidable disease progression and reduced treatment efficacy.^35^ Comparably substantial diagnostic capacity constraints and subsequent wait times have been predicted for other European countries, such as Sweden^42^ and England.^43^ First real‐world investigations from US specialty centers also report a significantly longer diagnostic process for confirmatory biomarker testing with CSF and/or amyloid PET as opposed to the use of a single, for example, blood biomarker test (Hofmann et al., CTAD conference 2025). Within Europe, these circumstances might provoke a debate about acceptable wait times, which will involve not only organizational but also ethical aspects.^42^, ^44^

Early and accurate diagnosis of AD also has downstream economic benefits. ATT are probably most effective the earlier they are initiated.^11^ Timely initiation of treatment may not only delay disease progression, but also reduce caregiver burden, and postpone institutionalization, each of which carries substantial cost implications for public health‐care systems. To realize these benefits, increased public awareness and corresponding policy alignment are essential. It is strongly recommended to update reimbursement frameworks as well as invest in a corresponding infrastructure to support the use of blood biomarkers and digital cognitive testing tools in routine care.

## 7 EU'S GENERAL DATA PROTECTION REGULATION: POTENTIAL RISK FOR INTERNATIONAL RESEARCH COLLABORATION

While digital and blood biomarker–based diagnostics offer clinical and economic advantages, their implementation must also navigate the regulatory landscape, particularly the EU's General Data Protection Regulation (GDPR). On the one hand, GDPR provides essential safeguards for patient privacy, ensuring that sensitive health data are handled with transparency and consent. This fosters public trust and ethical integrity in the deployment of novel diagnostic technologies. On the other hand, GDPR can introduce significant administrative and logistical burdens, especially for cross‐border data sharing, real‐world evidence generation, and AI model training. These constraints may affect international cooperation, slow innovation, and complicate the integration of digital platforms into routine care.

A balanced approach is needed, one that upholds data protection while enabling responsible and interoperable use of health data to support the overarching goal of a timely and cost‐effective AD diagnosis for everyone.

## 8 EUROPEAN STAFFING IN DEMENTIA CARE: NEED FOR MODERNIZATION AND INTERDISCIPLINARY COLLABORATION

The successful implementation of ATT at a larger scale in clinical routine will also place high personnel demands. Provided that the relevant tasks—from patient screening and thorough education to therapy administration and monitoring—are distributed efficiently and, where possible, standardized, not all these requirements have to be met by the traditional roles of either physicians or nurses themselves. The US serves as an example in which nurse practitioners and physician assistants have been successfully integrated into neurological care, including ATT. Europe might be well advised to also diversify its medical professional staffing to alleviate the burden on existing dementia specialists; increase patient flow workforce; as well as ensure comprehensive, sufficient, and high‐quality care.^45^

Besides classical neurologists and psychiatrists, types of physicians that could manage treatment include geriatricians/geriatric psychiatrists, neuropsychiatrists, and behavioral neurologists, all of which have experience in diagnosing AD as well as detecting relevant differential diagnoses such as depression or psychosis. So‐called memory boards, based on the model of tumor boards in oncology, should be established, including colleagues from neurology, psychiatry, radiology, and further disciplines with the goal to discuss complex diagnostic and therapeutic decisions during regular meetings.

In this context, a specifically German health‐care system aspect appears questionable. Here, historically, AD diagnosis and treatment have been primarily conducted in psychiatric clinics. However, the clinical AD focus is now quickly shifting from symptom control to early detection and disease‐modifying treatment, requiring a biomarker‐based diagnosis, genetic testing (*APOE*), intravenous infusions, continued MRI monitoring, as well as handling of potentially severe side effects such as ARIAs and infusion‐related reactions—all aspects that are quite new to the field of psychiatry and in places might exceed existing infrastructure, capacities, and competences. Thus, it is clearly recommended that especially German neurologists become more involved in the dementia field and that more interdisciplinary settings are developed to provide timely, safe, and holistic AD patient care.

## 9 SAFETY IN ANTI‐AMYLOID THERAPY: COGNITIVE MONITORING, MRI SURVEILLANCE, AND DIGITIZATION AS KEY EUROPEAN WEAKNESSES

ATT represents a paradigm‐shifting therapy that necessitates complex and standardized coordination to secure high‐quality care and patient safety. ATT initiation beforehand requires a thorough, individualized, and substance‐specific (i.e., lecanemab versus donanemab) patient counseling about the expectable benefit–risk ratio as well as practical aspects. In the phase 3 clinical trials,^11^, ^20^ lecanemab and donanemab slowed cognitive decline in early symptomatic AD by ≈ 30%. The potential treatment effect must be weighed against most relevant side effects of ATT, that is, ARIA and infusion‐related reactions, both of which typically occur early during treatment. Importantly, the risk of symptomatic ARIA is relevantly higher in patients with mild dementia compared to those with MCI.^3^ Thus, starting treatment as early as possible is a goal aimed not only at maximizing efficacy but also at ensuring safety.

A distinctive feature of the EMA labels for ATT is the general exclusion of certain patient subgroups, such as *APOE* ε4 homozygotes and patients on anticoagulant medication. On the one hand, this may be reasonable especially given the fact that *APOE* ε4 carrier status is the most important risk factor for ARIA.^11^, ^20^ On the other hand, according to first real‐world experience with lecanemab from the United States, these patients can actually be treated safely (EISAI company, AAIC 2025), making the EMA decision increasingly hard to justify.

**FIGURE 2:**
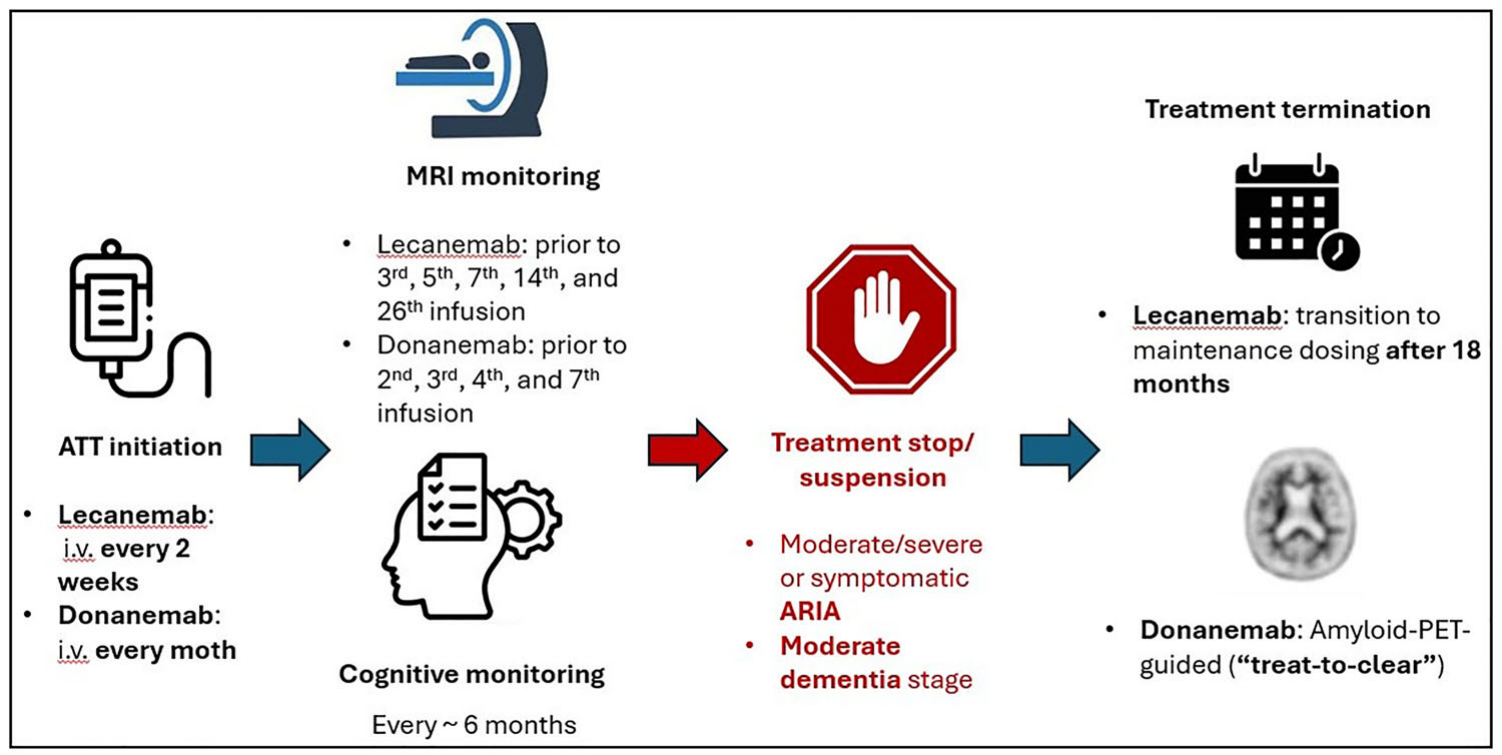
Patient management during ATT including MRI monitoring for ARIA. After treatment start with either lecanemab or donanemab, MRI and cognitive monitoring are regularly performed according to protocol. ATT should be suspended or even stopped prematurely in case of moderate to severe or symptomatic ARIAs as well as in moderate stages of dementia. Otherwise, patients on lecanemab typically transition to maintenance dosing after 18 months of treatment, whereas patients on donanemab end treatment once they achieved amyloid clearance by PET (typically after 12–18 months of treatment). ARIA, amyloid‐related imaging abnormalities; ATT, amyloid‐targeting treatment; i.v., intravenous (infusion); MRI, magnetic resonance imaging; PET, positron emission tomography.

In any case, an effective ATT monitoring strategy accordingly comprises sufficient capacity for cognitive testing, (urgent) MRI and emergency services, alongside a well‐developed digital infrastructure—domains in which Europe faces persistent gaps. Figure 2 summarizes key steps for safety management during ATT.

Tracking of cognitive status during treatment is critical for assessing therapeutic benefit as well as determining the time point for therapy discontinuation at moderate dementia stages, when efficacy is uncertain. Beside the already mentioned, limited access to neuropsychological services across Europe, importantly, there is no consensus yet on which cognitive instruments should guide clinical monitoring. While the CDR, MoCA, and Mini‐Mental State Examination (MMSE) are commonly used, their sensitivity and functional orientation vary. Further, MoCA may be particularly suited as a cognitive screening instrument for early stages of disease as, especially compared to the MMSE, it shows significantly less of a ceiling effect.^46^ In any case, harmonization of cognitive testing protocols is urgently required to ensure consistent and scalable cognitive monitoring as an all‐important outcome.

MRI capacity is uneven across Europe, with countries like the UK facing significant shortages. AI‐based tools may help support neuroradiologists by automating ARIA detection and quantification in the future.^47^ Nevertheless, dedicated personnel training programs for ARIA assessment will be vital to ensure safe and uniform imaging practices.

Emergency preparedness also must be embedded into treatment workflows. This includes establishing on‐call systems for after‐hours clinical support, protocols for rapid response to adverse events, and alerts for contraindicated medications such as thrombolytic agents. These safeguards are especially important in decentralized or outpatient settings in which immediate specialist access may be limited.

Electronic health records (EHR) remain underused and fragmented across European health‐care systems. Yet in the era of new AD treatments, EHR could play a transformative role by automating infusion scheduling, MRI surveillance, and longitudinal cognitive tracking. Investment in interoperable digital infrastructure is essential to streamline care and reduce administrative burden. Certain state‐of‐the‐art software products^48^ that are uniquely tailored for ATT and constantly evolving, have the potential to not only fundamentally simplify and improve clinical care, but also to connect real‐world data (RWD) collection with clinical registry digital infrastructures and/or scientific databases.

## 10 REAL‐WORLD DATA AND CLINICAL REGISTRIES: AIM FOR HARMONIZATION

As ATT transition from clinical trials to routine care, RWD registries are essential to monitor safety, effectiveness, and equity of access. These registries not only support post‐marketing surveillance but also enable learning health systems that can adapt and improve care delivery over time. In the United States, two major initiatives lead the way. The Alzheimer's Network for Treatment and Diagnostics (ALZ‐NET),^49^ coordinated by the international Alzheimer Association, collects longitudinal clinical and safety data from patients treated with ATT. It serves as a central platform for post‐marketing surveillance and research. In parallel, the Coverage with Evidence Development program of the Centers for Medicare & Medicaid Services requires data collection as prerequisite for ATT reimbursement.

In Europe, clinical registry efforts are growing in a more fragmented way. Essentially, efforts to join large international or global registries conflict with those to establish and promote national registries (Table 3). The International Registry for Alzheimer's Disease and Other Dementias (InRAD)^50^ provides a global, practice‐based platform for harmonized RWD collection. InRAD includes both treated and untreated patients and emphasizes standardization through a defined minimum and extended dataset, enabling cross‐country comparisons and collaborative research. Globally, Gates Ventures’ Alzheimer's Disease Data Initiative (ADDI) supports open‐access data platforms and interoperability standards to accelerate research and improve care. ADDI's emphasis on data sharing and harmonization complements efforts like ALZ‐NET and InRAD, helping to build a cohesive international registry ecosystem. Further, the European platform for neurodegenerative diseases with its main mission to remove barriers concerning data sharing and collaboration is pursuing scientific goals beyond immediate therapy monitoring, too.

**TABLE 3:** Several examples for ATT post‐approval registries in Europe. Columns provide more detailed information on the possibility for international data sharing, scientific and clinical applications, as well as relevance for reimbursement by insurance companies.

Registry	Cross‐country data sharing	Research use	Clinical use	Reimbursement
**International**				
Post‐Authorization Safety Study (PASS)/ Controlled Access Program (CAP)	No (missing integration)	Relevance unclear	Focus on pharmacovigilance	Required
International Registry for Alzheimer's Disease and Other Dementias (InRAD)	Possible through emphasis on standardization (minimum and extended dataset)	Strong focus, free‐to‐use data platform	Also untreated AD patients	Independent
Gates Ventures’ Alzheimer's Disease Data Initiative (ADDI)	Focus on data sharing and harmonization, complements efforts like ALZ‐NET and InRAD	Open‐access scientific data platform	Subordinate	Independent
**National**				
**Germany**				
DZNE Describe Therapy	No (national)	Strong focus, data available on demand	Only treated patients	Independent
German national dementia registry (DNG)	Not planned	Strong focus, data available on demand	Also untreated AD patients	Independent
NeuroTransData	No (national)	None	Outpatient setting	Independent
**France**				
Banque Nationale Alzheimer (BNA)	No, but currently aiming at integration with national health data systems	Strong focus, data available on demand	Captures demographic, diagnostic, and care‐related data	Independent
**UK**				
Dementias Platform UK	Yes, focus on data sharing	Strong focus, data available on demand	Also untreated AD patients	Independent
BARBARA	Generally possible	Data available on demand	Biomarker focus	Independent
**Netherlands**				
A Personalized Medicine Approach for Alzheimer's Disease (ABOARD)	Generally possible (particularly EU countries)	Strong focus, data available on demand	Integrating patient‐reported outcomes and digital cognitive assessments	Independent

Abbreviations: AD, Alzheimer's disease; EMA, European Medicines Agency; EU, European Union.

On the national level, for example in Germany, several initiatives are underway, including the research‐focused German Center for Neurodegenerative Diseases (DZNE) Describe Therapy registry; the German National Dementia Registry operated by the German Network for Memory Clinics (DNG); and the NeuroTransData registry, focused on outpatient care. In France, the *Banque Nationale Alzheimer* collects data from > 600 memory centers, thereby capturing diagnostic, demographic, and care‐related data. In the UK, the Dementias Platform UK provides infrastructure for data sharing and cohort integration, while the BARBARA initiative focuses on biomarker‐based registry development and translational research. Another UK‐based registry, ACCESS AI, focuses on clinical–practical aspects, such as the development of ultra‐fast, AI‐driven MRI techniques. The Dutch ABOARD registry establishes a nationwide, participant‐centered infrastructure that integrates patient‐reported outcomes, clinical data, and digital cognitive assessments to longitudinally map AD trajectories, thereby enabling stratified treatment approaches and accelerating the implementation of personalized medicine in routine care.

In parallel to these European endeavors, the manufacturers of lecanemab and donanemab on the instructions of EMA are launching a Controlled Access Program (CAP) on national levels. Further, a Post‐Authorization Safety Study (PASS) for both substances is in preparation and expected to be published in 2026 as part of regulatory obligations. While these initiatives are important for pharmacovigilance, they are limited in scope. The CAP restricts access to treatment through narrow eligibility and monitoring criteria, while the PASS collects only a minimal dataset and is not designed for broader scientific utility. Moreover, these programs are not integrated with national or international registries, limiting their value for comparative effectiveness, research, health system planning, or equity monitoring.

Europe should prioritize harmonization of registry datasets, ideally aligned with international standards based on broad consensus among key stakeholders, including clinicians, academia, patient advocacies, non‐governmental organizations and industry. Clear task allocation among registries is needed to avoid redundancy and ensure comprehensive coverage. Integration with digital health infrastructure, including EHR and cognitive tools, will be essential to streamline data capture and reduce clinician burden. A coordinated European strategy, linked to global platforms like ALZ‐NET, InRAD, and ADDI, would not only enhance post‐marketing surveillance but also accelerate learning, innovation, and equitable access in AD care.

## 11 CALL TO ACTION: ALIGNING SCIENCE, POLICY, AND PRACTICE FOR EQUITABLE AD CARE IN EUROPE

**FIGURE 3:**
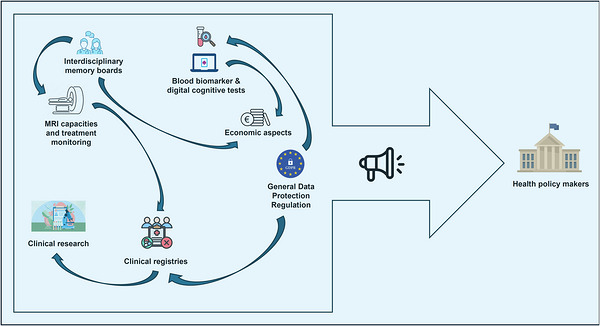
Major health policy–related obstacles and opportunities considering the successful clinical implementation of ATT in Europe. Incorporation of blood biomarker tests and digital cognitive screening tools in clinical routine would allow for an early diagnosis that is accessible for the broader population. This could result in significant economic savings that might be further increased by a targeted personnel diversification to alleviate the burden on existing dementia specialists as well as to foster interdisciplinary collaboration. The latter also represents an important prerequisite for safe therapy administration and monitoring; however, it is imperative that European countries increase their MRI capacities, too. Further, the harmonized collection of clinical, cognitive, and imaging data in interoperable clinical registries will be vital for the deployment of anti‐amyloid treatment across Europe, ultimately leading to effective clinical research. Especially the latter step might be endangered by the EU's General Data Protection Regulation, which is complicating the integration of novel diagnostic platforms into routine care and hindering cross‐border data sharing. Therefore, there is an urgent call for health policy makers to modernize European diagnostic and treatment pathways to ensure an equitable and timely access to disease‐modifying therapies for all AD patients that might benefit. AD, Alzheimer's disease; *APOE*, apolipoprotein E; ATT, amyloid‐targeting treatment; MRI, magnetic resonance imaging.

Europe stands at a pivotal moment in the fight against AD. The approval of the first ATT has redefined what is possible, but without bold, coordinated action, these advances risk benefiting only a privileged few. We call on European clinicians, research institutions, and policy makers to urgently modernize diagnostic and treatment pathways (Figure 3).

The position taken by some medical societies discouraging biomarker testing in individuals with early clinical AD is no longer tenable in light of current evidence. Primary care providers must be empowered to play a central role in early detection, supported by digital cognitive tools and validated blood biomarkers. To ensure safe and effective treatment monitoring, Europe must invest in MRI capacity, adopt harmonized cognitive assessment protocols, and expand digital infrastructure. The proliferation of fragmented registries must give way to coordinated, interoperable data systems that support both patient safety and scientific discovery at a global scale. The tools are here.

The current situation opens a unique opportunity to intentionally create a synergism between clinical care and research via standardization of outcome measures as well as establishment of regular biofluid and imaging sampling with the aim to address the many unanswered questions related to real‐world ATT application as determined and quickly as possible. The science is ready. What remains is the political and professional will to act. Europe must not delay.

## CONFLICT OF INTEREST STATEMENT

A.H. has received research funding from Novo Nordisk, hospitality from Eli Lilly, and has collaborated with Biogen on a research project. R.P. has received honoraria for advisory boards and speaker engagements from Roche, EISAI, Eli Lilly, Biogen, Janssen‐Cilag, AstraZeneca, Schwabe, Grifols, Novo Nordisk, and Tabuk, and is a shareholder of Medotrax GmbH (owner of BraincheX) and Vistim Ltd., and the Founding Chairman of the Board of the International Registry for Alzheimer Disease and Other Dementias (InRAD). Author disclosures are available in the Supporting Information.

## Supporting information



Supporting Information
